# An Inaugural Address, Delivered at the Opening of the Norfolk and Norwich Hospital Museum, Sept. 1845

**Published:** 1846-01

**Authors:** 


Art. XIII.—*An Inaugural Address, delivered at the Opening of the Norfolk and Norwich Hospital Museum,* Sept. 10, 1845. By J. C. Crosse, m.d., f.r.s., the Senior Surgeon to the Hospital. *Norwich,* 1845. 8vo, pp. 28.

We can pay this little book no higher compliment than to say, it is a fitting memorial of the event it celebrates. Its author is one of the principal founders of the splendid Institution which now enriches and adorns the Medical School at Norwich; and the character of this Address proves that its literary inauguration could not have been intrusted to an abler pen. Dr. Crosse gives a delightful account of the many great men of our profession who have illustrated Norwich, including the names of Kaye or Caius, Sir Thomas Browne, Howman, Benjamin Gooch, William Donne, Alderson, Yelloly, Wright, Rigby, Martineau, and Dalrymple—the last alone surviving. We doubt not that those who heard this eloquent Address, felt that there were yet giants in the land; and all who know Dr. Crosse's writings are aware that the future historian of Norwich will have to place his name not last in the roll of the medical worthies who give lustre to that ancient city. We regret that our circumscribed space prevents us doing justice to Dr. Crosse's Prolusion. We can only introduce, by way of specimen, a fragment of the sketch he has given of Mr. Dalrymple.

"All unbecoming competition Mr. Dalrymple was a stranger to, for his ardent love of the calling he had embraced, and so successfully pursued, was the powerful and the only needed stimulus to his exertions. Whilst practising in this city, he constantly showed himself more jealous of his reputation than of his purse; he left the public to find out his qualifications, and required that it should seek to reward him, being not in the pursuit of wealth, like one possessed of mediocrity of talent, but in pursuit of the science of his profession and of his own continual improvement in that science. It appears that Mr. Dalrymple was many years resident in this city ere his talents were adequately appreciated, and longer before he became connected with this Hospital. His rise was the slower, but perhaps the more secure, from his delicacy on certain professional points, and his deference to certain customs which help to regulate our profession, and which if young men do not respect, they have no longer any guide but their passions. People are led so much more by their prejudices and feelings, than by reason and good judgment, that the art of rising in practice consists as much in *knowing how to please*, as *how to cure diseases;* it is not surprising that Mr. Dalrymple, who so well understood *both* these, should have ultimately gained the most extensive employment and the highest reputation in his profession; no man knew better how to unlock the affections of those patients whom he once attended, and he opened all the avenues to their understandings by the most refined eloquence. . . . Who is there that knew much of him, and did not applaud that great sensitiveness for his patients, which led him to feel interested in their comforts and well-doing, to his own distress and injury? or has not been captivated by his eloquence, his ready wit, and his extensive information, available upon all the general topics that engage the attention of the most polished society, and which imparted a charm to his conversation that raised him above all ordinary men? Amidst the honorable strife inseparable from our calling and under circumstances which always placed me in unequal competition, no living being has ever heard from my lips a word respecting Mr. Dalrymple, that was not in strict accordance with this public statement; I have no winged words of flattery for this occasion, and there is no fear of my language surpassing the subject of my discourse, as regards his professional abilities and character."

